# Rehabilitation Applications Based on Behavioral Therapy for People With Knee Osteoarthritis: Systematic Review

**DOI:** 10.2196/53798

**Published:** 2024-05-02

**Authors:** Dian Zhu, Jianan Zhao, Mingxuan Wang, Bochen Cao, Wenhui Zhang, Yunlong Li, Chenqi Zhang, Ting Han

**Affiliations:** 1 School of Design Shanghai Jiao Tong University Shanghai China; 2 Department of Design Jiangxi Science and Technology Normal University Shanghai China; 3 Institute of Medical Robotics Shanghai Jiao Tong University Shanghai China

**Keywords:** knee osteoarthritis, digital application, behavioral therapy, behavior change therapy, cognitive behavioral therapy

## Abstract

**Background:**

The development of digital applications based on behavioral therapies to support patients with knee osteoarthritis (KOA) has attracted increasing attention in the field of rehabilitation. This paper presents a systematic review of research on digital applications based on behavioral therapies for people with KOA.

**Objective:**

This review aims to describe the characteristics of relevant digital applications, with a special focus on the current state of behavioral therapies, digital interaction technologies, and user participation in design. The secondary aim is to summarize intervention outcomes and user evaluations of digital applications.

**Methods:**

A systematic literature search was conducted using the keywords “Knee Osteoarthritis,” “Behavior Therapy,” and “Digitization” in the following databases (from January 2013 to July 2023): Web of Science, Embase, Science Direct, Ovid, and PubMed. The Mixed Methods Assessment Tool (MMAT) was used to assess the quality of evidence. Two researchers independently screened and extracted the data.

**Results:**

A total of 36 studies met the inclusion criteria and were further analyzed. Behavioral change techniques (BCTs) and cognitive behavioral therapy (CBT) were frequently combined when developing digital applications. The most prevalent areas were goals and planning (n=31) and repetition and substitution (n=27), which were frequently used to develop physical activity (PA) goals and adherence. The most prevalent combination strategy was app/website plus SMS text message/telephone/email (n=12), which has tremendous potential. This area of application design offers notable advantages, primarily manifesting in pain mitigation (n=24), reduction of physical dysfunction (n=21), and augmentation of PA levels (n=12). Additionally, when formulating design strategies, it is imperative to consider the perspectives of stakeholders, especially in response to the identified shortcomings in application design elucidated within the study.

**Conclusions:**

The results demonstrate that “goals and planning” and “repetition and substitution” are frequently used to develop PA goals and PA behavior adherence. The most prevalent combination strategy was app/website plus SMS text message/telephone/email, which has tremendous potential. Moreover, incorporating several stakeholders in the design and development stages might enhance user experience, considering the distinct variations in their requirements. To improve the efficacy and availability of digital applications, we have several proposals. First, comprehensive care for patients should be ensured by integrating multiple behavioral therapies that encompass various aspects of the rehabilitation process, such as rehabilitation exercises and status monitoring. Second, therapists could benefit from more precise recommendations by incorporating additional intelligent algorithms to analyze patient data. Third, the implementation scope should be expanded from the home environment to a broader social community rehabilitation setting.

## Introduction

Knee osteoarthritis (KOA) is a prevalent musculoskeletal disorder that ranks among the primary contributors to disability [1,2]. Possible long-term ramifications encompass diminished levels of physical activity (PA), the development of body dysmorphic disorder, compromised sleep patterns, depressive symptoms, and the onset of disability [3,4]. In recent times, there has been a notable shift in the approach to treating KOA, with a greater emphasis on nonpharmacologic interventions. This change is supported by evidence indicating that nonpharmacologic treatments are more effective in delivering sustained symptom alleviation and in delaying or even preventing functional deterioration [5,6]. The primary nonpharmacological interventions for KOA include educational programs, PA interventions, and weight management strategies [3]. Patient initiation and adherence to these treatments are essential factors for achieving effective symptom control [7]. Traditional nonpharmacological interventions, however, require professional guidance to achieve the desired results, which is associated with high costs and unequal health care resources [8].

Digital health interventions have the potential to offer widespread, cost-effective, readily available, and easily expandable patient education and self-management interventions for individuals with KOA [9-11]. Several research investigations have been carried out to substantiate their efficacy in rehabilitating musculoskeletal problems. For instance, digital health interventions have been found to be successful in decreasing pain, improving functionality, and promoting the self-management of musculoskeletal pain syndromes [12,13]. Significant increases in adherence have also been observed throughout the mid-term follow-up [14]. These systematic evaluations have focused on summarizing various techniques for digital health or intervention effectiveness in relation to health outcomes [15,16]. However, digital interventions do not always provide desirable outcomes. Providing guidance on the ideal dosage required to achieve significant benefits or disclosing the elements of effective digital health treatments is challenging owing to the variations in interventions and the insufficient information in interventions [15]. In recent years, it has been discovered that theory-driven interventions can help organize the content of digital interventions, resulting in improved health outcomes [17-19].

A growing number of studies have used the behavioral psychology theoretical framework in digital format [20]. Compared to generic digital interventions, behavioral therapy-based digital interventions are significantly more effective at relieving pain, improving physical dysfunction, and increasing self-efficacy in patients with KOA [21,22]. Physiotherapists use scalable interventions along with some digital tools to enhance treatment adherence [16,23]. The concept of behavioral therapy (BT) incorporates various therapeutic approaches, including behavioral change techniques (BCTs), dialectical behavioral therapy (DBT), and cognitive behavioral therapy (CBT). It has been used to aid complex intervention designs that include facilitating the adoption of behavior change, promoting behavioral compliance, sustaining behavioral change, and preventing behavioral relapse [24]. Previous studies have employed BCTs in combination with digital interventions among individuals with musculoskeletal pain [25]. These studies have reported the efficacy of such interventions in facilitating the transition of patients from a sedentary lifestyle to an active one [21,22,26]. Multiple studies have demonstrated that the integration of CBT with standard care yielded noteworthy outcomes in the management of KOA. Specifically, the implementation of CBT interventions resulted in a considerable reduction in pain levels and an improvement in insomnia symptoms when compared to the utilization of standard care alone, as indicated by previous investigations [27,28].

Currently, there are evaluations investigating the rehabilitative impacts of digitalization in KOA and highlighting the significance of behavioral theory in some applications [19]. Nevertheless, there is a shortage of thorough exposition of the behavioral theory in digital applications, as well as an absence of an assessment of the suitability of these applications from the patient’s point of view. Thus, this review offers a methodical and thorough examination of digital applications rooted in behavioral therapy. It shifts the focus of digital applications from mere practical usability to providing support for behavioral change theories. Additionally, it meticulously analyzes the functional reasoning behind various products, thereby serving as a comprehensive guide for designing future digital interventions. Hence, the objectives of this review are to (1) provide a concise overview of the existing landscape of digital behavioral therapy applications for individuals diagnosed with KOA and examine the potential of digital applications in augmenting the rehabilitation process for KOA patients, and (2) present a comprehensive analysis of the underlying psychological theories, fundamental mechanisms, design methodologies, typical attributes, efficacy of treatment outcomes, and patient preferences pertaining to this particular mode of recovery intervention.

## Methods

### Registration

This review has been registered in the International Prospective Register of Systematic Reviews (PROSPERO; registration number: CRD42023430716). Furthermore, the PRISMA (Preferred Reporting Items for Systematic Reviews and Meta-Analyses) guidelines have been applied (Multimedia Appendix 1) [29].

### Search Strategy

Literature searches were conducted in 5 databases: Web of Science, Embase, Science Direct, Ovid, and PubMed. The selection of these databases was based on their provision of comprehensive access to full-text journals and conference proceedings pertaining to prominent conferences and meetings focused on digital technology and medicine.

To locate relevant articles, we conducted a search by filtering papers based on 3 primary categories of MeSH (Medical Subject Headings) terms: “Knee Osteoarthritis,” “Behavioral Therapies,” and “Digitization.” According to MeSH terminology, “Behavioral Therapy” is divided into “Behavioral Therapy,” “Cognitive Behavioral Therapy,” and “Dialectical Behavioral Therapy.” In this review, in order to understand the categorization of all behavioral therapies, we collected information on the subcategories of these 3 categories related to behavior. In relation to the subject of “digital” content, we gathered relevant material from the report by Safari et al [19] on digital literature, encompassing topics, such as “Telehealth,” “Email,” “Smartphone,” “Computer Systems,” “Digital Technologies,” and “Mobile Applications,” and other forms of digitization. Table 1 illustrates sample search strategies used for the PubMed digital library. The article titles, keywords, and abstracts were searched. Similar search strategies were applied to the remaining 3 databases. Relevant articles published between January 2013 and July 2023 were gathered. We included journal papers and peer-reviewed conference proceedings. Only articles written in English were considered.

**Table 1:** Literature search strategy.

MeSH^a^	Boolean logic search strings
Knee Osteoarthritis	“Knee Osteoarthritides” OR “Knee Osteoarthritis” OR “Osteoarthritis of Knee” OR “Osteoarthritis of the Knee”
Behavior Therapy	“Behavior Therapies” OR “Behavior Treatment” OR “Conditioning Therapy” OR “Conditioning Therapies” OR “Behavior Change Techniques” OR “Behavior Change Technique” OR “Behavior Modification” OR “Behavior Modifications” OR “Dialectical Behavior Therapies” OR “Cognitive Behavioral Therapies” OR “Cognitive Therapy” OR “Cognitive Behavior Therapy” OR “Cognitive Psychotherapy” OR “Cognition Therapy” OR “Cognitive Behavior Therapies” OR “Cognitive Behavior Therapy”
Digitization	“Telemedicine” OR “Mobile Health” OR “Telehealth” OR “ehealth” OR “mhealth” OR “Email” OR “E-mail” OR “Mobile” OR “Smartphone” OR “smart-phone” OR “smart telephone” OR “Tablet” OR “cell” OR “hand-held” OR “Cell Phone” OR “handheld” OR “Remote Consultation” OR “Teleradiology” OR “Telenursing” OR “Computer Systems” OR “Computer-Assisted Instruction” OR “Internet” OR “web” OR “computer” OR “Digital Technologies” OR “APP” OR “Social Media” OR “Internet-Based Intervention” OR “Mobile Application” OR “Mobile App” OR “Smartphone App” OR “Portable Software Application”

^a^MeSH: Medical Subject Headings.

### Eligibility Criteria

The authors DZ and JZ were assisted in the literature search by an experienced librarian well versed in medical database searching. This literature review was guided by the question of how behavioral therapies can be integrated with digital applications in the rehabilitation of patients with KOA. On this basis, we anticipated that this review would (1) generalize and summarize the digital applications used in behavioral therapy, and (2) describe the overall research status and research trends of these digital applications.

#### Inclusion Criteria

The inclusion criteria were as follows: (1) adult participants (age ≥18 years) with KOA diagnosed by self-reported symptoms or imaging; (2) patients had access to digital applications; (3) any form of intervention or treatment based on the inclusion of at least one behavioral treatment was delivered through any digital application (eg, website or app) within any time frame; and (4) the described interventions were compared to waiting list control (no intervention) or alternative (standard) delivery modalities (eg, face-to-face approaches, classroom-based approaches, and printed materials or handouts), nondigital self-management interventions, and noninteractive digital interventions (eg, web pages with flat copies).

#### Exclusion Criteria

The exclusion criteria were as follows: (1) patients with KOA were not included; (2) nondigital interventions were assessed; (3) behavioral therapies were not included; (4) research protocols, reviews, conceptual articles, case studies or discussion papers, and conference abstracts; (5) market research; (6) digitization was not designed for the recovery process; (7) text was not written in English; and (8) duplicate reports of the same study from different sources.

### Data Extraction

Data relevant to the purpose of the study were extracted independently by the authors MW, WZ, and BC, and any misunderstandings and disagreements were resolved through negotiation. Extracted data included study context details, study population, and digital application details. In more detail, the template included the following categories: (1) basic information (author, year, origin, study population, sample size, presence of a physiotherapy intervention, and duration of the intervention); (2) digital application details (digitalization of behavioral therapy, interactive device function, study outcomes, and application deficiencies); and (3) type of study (randomized controlled trial, cohort experiment, experimental protocol, and qualitative study).

### Quality Assessment

The Mixed Methods Assessment Tool (MMAT) was used to evaluate the methodological quality of the included studies [30]. This tool was initially created in 2006 through a comprehensive analysis of systematic evaluations that integrated qualitative and quantitative evidence. In 2018, a revised version of the MMAT was developed by assessing its usefulness, reviewing key assessment tools in the literature, and conducting a modified e-Delphi study involving methodology experts to determine the essential criteria (Multimedia Appendix 2). The MMAT evaluates the caliber of research employing qualitative, quantitative, and mixed approaches. The primary emphasis is on methodological standards, which encompass 5 fundamental quality criteria for 5 distinct study designs: (1) qualitative, (2) randomized controlled, (3) nonrandomized, (4) quantitative descriptive, and (5) mixed methods.

## Results

### Overview

**Figure 1:**
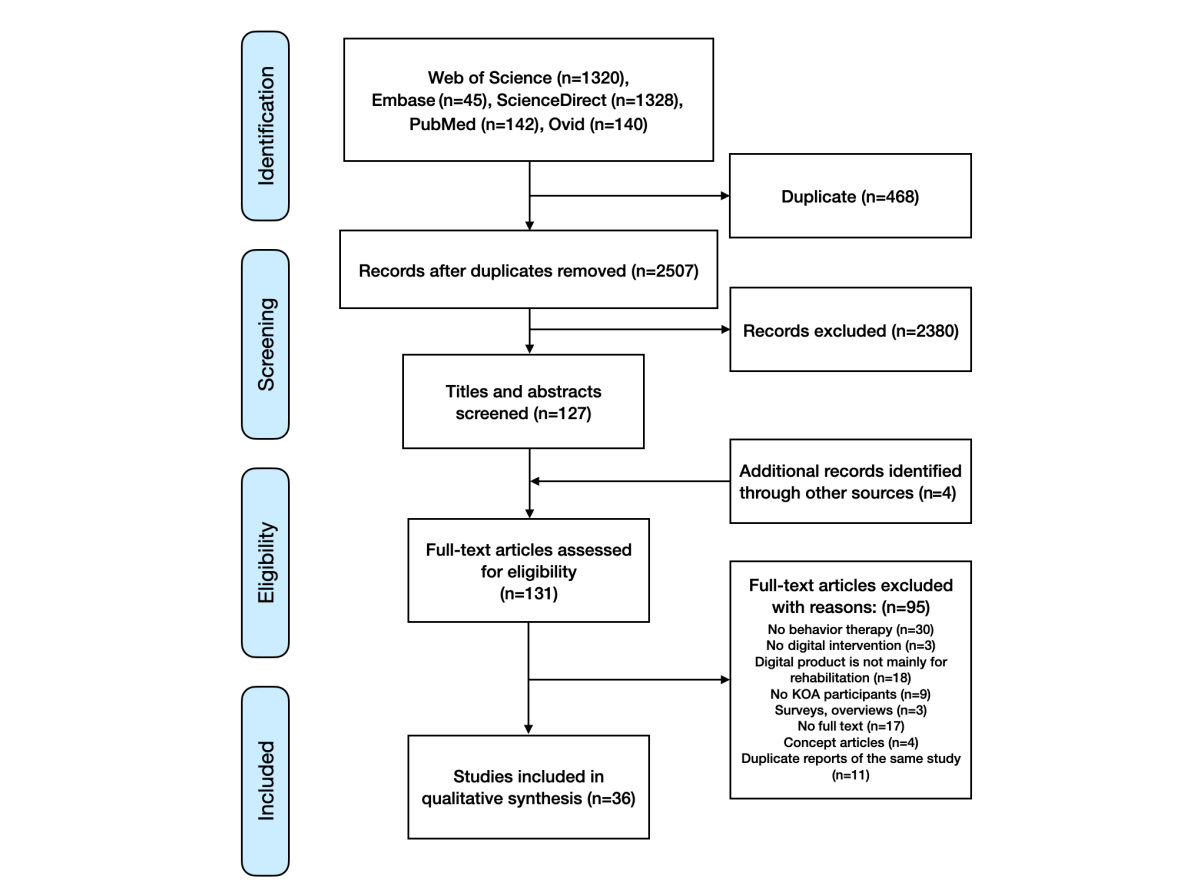
PRISMA (Preferred Reporting Items for Systematic Reviews and Meta-Analyses) flowchart. KOA: knee osteoarthritis.

Figure 1 provides a summary of the outcomes at various phases of article selection. The search results of the databases are provided in Multimedia Appendix 3. Based on the search strategy, 2975 articles were found initially, and 2507 articles remained after title and abstract screening and removal of duplicates. Next, full-text articles were chosen based on the inclusion and exclusion criteria. We included a total of 131 articles explicitly related to digital behavioral therapy for KOA. Moreover, 4 articles were identified following a manual search of the references for articles that were cited. Final consideration was given to 36 articles for systematic evaluation (Table 2).

**Table 2:** Information on the included studies.

Author	Year	Country	Population	Sample size	Physiotherapist	Duration	Digital forms	Experiment	Quality
Bossen et al [31]	2013	Netherlands	KOA^a^/HOA^b^	20	Yes	6-12 weeks	Internet platform + SMS text message/telephone	Nonrandomized pilot study	4
Rini et al [32]	2016	United States	OA^c^	113	No	9-11 weeks	App + “virtual coaching”	Randomized controlled trial	5
Pearson et al [33]	2016	United Kingdom	KOA/HOA	200	No	—^d^	Internet website	Mixed methods research	2
Bennell et al [34]	2017	Australia	KOA	168	Yes	24 weeks	Internet platform + SMS text message/telephone	Randomized clinical trial	5
Bennell et al [11]	2017	Australia	KOA	148	Yes	24 weeks	Internet platform + SMS text message/telephone	Randomized clinical trial	5
Li et al [35]	2017	Canada	KOA	34	Yes	8 weeks	Electronic equipment + telephone	Randomized controlled trial	4
Lawford et al [36]	2018	Australia	KOA	148	Yes	12-36 weeks	PainCOACH software + email	Randomized controlled trial	5
Button et al [37]	2018	United Kingdom	KOA	49	Yes	12 weeks	Online course	Qualitative research	4
Mecklenburg et al [38]	2018	United States	KOA	162	Yes	12 weeks	Hinge Health app + wearable	Randomized controlled trial	5
Kline et al [39]	2019	United States	TKR^e^	100	Yes	—	Online course + wearable	Randomized controlled trial protocol	N/A^f^
Nelligan et al [40]	2019	Australia	KOA	12	No	24 weeks	SMS text message application + messaging interactions	Qualitative research	5
Pelle et al [41]	2019	Netherlands	KOA/HOA	427	No	12-24 weeks	Bart app	Randomized controlled trial	5
Bailey et al [42]	2020	United States	MD^g^	10,264	Yes	9 weeks	Hinge Health app + wearable motion sensors	Cohort study	4
Baker et al [43]	2020	United States	KOA	104	Yes	2 years	Teleconferencing (remote software)	Cohort study	4
Bennell et al [22]	2020	Australia	KOA/obesity	12	No	24 weeks	SMS text message application + messaging interactions	Randomized controlled trial	5
Fitzgibbon et al [44]	2020	United States	OA	203	Yes	8 weeks	F&S! and F&S! Plus + telephone	Comparative effectiveness test	4
Hinman et al [45]	2020	Australia	KOA	165	Yes	24 weeks	Internet website + SMS text message/telephone	Randomized controlled trial	5
Hinman et al [46]	2020	Australia	KOA	394	Yes	12 weeks	Internet website + video consulting	Randomized controlled trial protocol	N/A
Li et al [47]	2020	Canada	KOA	51	Yes	12 weeks	Electronic device + telephone/mail	Randomized controlled trial	4
Nelligan et al [48]	2020	Australia	KOA	16	Yes	—	Internet website + SMS text message/telephone	Qualitative research	2
Dunphy et al [49]	2021	United Kingdom	OA	59	Yes	12 weeks	Internet website	Two-arm parallel randomized controlled trial	5
Lindberg et al [50]	2021	Norway	OA	282	Yes	12 weeks	iCBT application + telephone	Randomized controlled trial protocol	N/A
Nelligan et al [21]	2021	Australia	KOA	206	Yes	24 weeks	Internet website + SMS text message/telephone	Randomized controlled trial	5
Pelle et al [51]	2021	Netherlands	KOA/HOA	214	No	26 weeks	Bart app	Randomized controlled trial	4
Rognsvåg et al [52]	2021	United Kingdom	KOA/TKR	4	Yes	—	iCBT application + telephone	Qualitative research	3
Bennell et al [53]	2022	Australia	KOA	88	Yes	24 weeks	Website + remote software	Randomized controlled trial protocol	N/A
Groves-Williams et al [54]	2022	Scotland	KOA	90	No	12-36 weeks	Internet website + SMS text message/telephone	Randomized controlled trial protocol	N/A
Hinman et al [55]	2022	Australia	KOA	182	Yes	14 weeks	App + SMS text message	Randomized controlled trial protocol	N/A
Östlind et al [56]	2022	Sweden	KOA/HOA	20	Yes	12 weeks	App + electronic device	Qualitative research	4
Östlind et al [57]	2022	Sweden	KOA/HOA	160	Yes	12 weeks	App+ electronic device	Randomized controlled trial	3
Whittaker et al [58]	2022	Canada	OA	30	Yes	4 weeks	Video conferencing + wearable + app	Randomized trial	4
Godziuk et al [59]	2023	Canada	KOA	53	Yes	12 weeks	Website + email	Cohort study	3
Lorbeer et al [60]	2023	Germany	KOA	241	Yes	1 year	Teleconferencing (remote software)	Randomized controlled trial	4
Scheer et al [61]	2023	United States	MD	4051	Yes	12 weeks	App + electronic device	Cohort study	4
Truong et al [62]	2023	Canada	OA	16	No	—	Video conferencing + wearable + app	Qualitative research	5
Weber et al [63]	2023	Germany	KOA/HOA	330	Yes	3 weeks	e-Exercise + online physiotherapy	Randomized controlled trial protocol	N/A

^a^KOA: knee osteoarthritis.

^b^HOA: hip osteoarthritis.

^c^OA: osteoarthritis.

^d^Data not available.

^e^TKR: total knee replacement.

^f^N/A: not applicable.

^g^MD: musculoskeletal disorder.

### Methodological Quality

Among the included studies, 12 met 100% of the quality assessment criteria, 15 fulfilled 60%-80% of the quality assessment criteria, and 2 met 40% of the quality assessment criteria (Multimedia Appendix 4 [11,21,22,31-63]). The remaining 7 studies could not be evaluated for their quality owing to the absence of results. Nevertheless, the application description portion involved in the studies was highly valuable for analysis.

### Digitalization of Behavioral Therapy

#### Behavior Change Therapy

The majority of digital applications for KOA rehabilitation are based on BCTs. BCTs (achieving objectives, setting goals, restructuring beliefs, and inducing acceptance) are applicable to addressing the central issues of initiating and maintaining PA [64]. The primary categories of BCTs used in the reviewed studies were based on the V1 Taxonomy of Behavior Change by Michie et al [65], which was devised by behavior change researchers [66]. The taxonomy comprises 93 distinct BCTs organized into 16 hierarchical structures and has been extensively used in the literature on behavior change (Table 3): (1) goals and planning; (2) feedback and monitoring; (3) social support; (4) shaping knowledge; (5) natural consequences; (6) behavioral comparisons; (7) associations; (8) repetition and substitution; (9) outcome comparisons; (10) rewards and threats; (11) regulation; (12) presuppositions; (13) identity; (14) intended consequences; (15) self-confidence; and (16) implicit learning.

**Table 3:** Behavioral change techniques in digital applications.

Cluster label and component behavioral change techniques		References
**1: Goals and planning**		
	1.1: Goal setting (behavior)	[11,31-37,39-47,49-52,54,58,59,61,62]
	1.2: Problem solving/coping planning	[35,39,44,54,63]
	1.4: Action planning	[11,21,31-37,43,46,48,52,53,60,63]
	1.5: Review of behavior goal(s)	[11,31,33,34,39,43,52]
	1.7: Review of outcome goal(s)	[11,34,58,62]
**2: Feedback and monitoring**		
	2.2: Feedback on behavior	[33,42,56,57]
	2.3: Self-monitoring of behavior	[31,32,36,39,42,44,47,55,57-59,62]
	2.4: Self-monitoring of the outcome of behavior	[33,47,58,61]
	2.6: Biofeedback	[11,33-35,37,39,42,47,56-58,61,62]
	2.7: Feedback on behavioral outcomes	[32,36]
**3: Social support**		
	3.2: Social support (practical)	[33,59]
	3.3: Social support (emotional)	[43,60]
**4: Shaping knowledge**		
	4.1: Instructions on how to perform a behavior	[21,32,42-44,46-50,52-54,56,59,61]
	4.2: Antecedents	[32,36,38]
**5: Natural consequences**		
	5.1: Health consequences	[32,36,39,41,42,50,51]
	5.4: Self-assessment of affective consequences	[33,43]
	5.5: Anticipated regret	[35,39,43,63]
**6: Comparison of behavior **		
	6.1: Modeling of behavior	[42,56]
	6.2: Social comparison	[33,53,60]
	6.3: Information about others’ approval	[32,36,42-44,49,53,58,59,61-63]
**7: Associations**		
	7.1: Prompts/cues	[11,21,22,31,34,37,40,43,45,48,50,54,55,59,60]
**8: Repetition and substitution**		
	8.1: Behavioral rehearsal/practice	[11,21,34,35,37,39-43,46,48,49,51,56,58,59,61-63]
	8.6: Generalization of a target behavior	[33,38,42]
	8.7: Graded tasks	[31,33,45,49,63]
**10: Reward and threat**		
	10.3: Nonspecific reward	[32,36]
**11: Regulation **		
	11.2: Regulate negative emotions	[33,38,42,43,50,52,59]
**12: Antecedents **		
	12.4: Distraction	[32]
**13: Identity **		
	13.1: Identification of self as a role model	[52]
**15: Self-belief **		
	15.1: Verbal persuasion to boost self-efficacy	[32,36,39]
**16: Covert learning**		
	16.2: Covert conditioning	N/A^a^
	16.3: Vicarious reinforcement	[43]

^a^N/A: not applicable.

In 31 studies, objectives and planning were mentioned, including goal setting (behavior), problem solving or coping planning, and reviewing behavioral or outcome goals. Among these factors, goal setting and action planning were shown to be the most prominent components within the area. A total of 26 applications included goal setting, which has a very broad definition in the taxonomy (setting goals defined according to the behavior or outcome to be accomplished) [11,31-37,39-47,49-52,54,58,59,61,62]. Applications created evidence-based, individualized, progressive home exercise plans; promoted increased general PA; and established short-term objectives. Moreover, 16 applications [11,21,31-37,43,46,48,52,53,60,66] contained action planning in which patients were asked or chose to perform activities until their pain tolerance was attained, based on which the patients prescribed their own individual therapeutic actions. Additionally, 5 applications [35,39,44,54,63] addressed problem solving and coping strategies encountered during rehabilitation by other individuals or physiotherapists. Furthermore, 9 applications contained a review of behavioral or outcome objectives [11,31,33,34,39,43,52,58,62], encouraging participants to monitor their progress and assisting them in identifying personal barriers and strategies for overcoming them.

A total of 19 investigations included various forms of feedback and monitoring, such as feedback on behavior, self-monitoring of behavior, biofeedback, self-monitoring of behavioral outcomes, and feedback on behavioral outcomes. Feedback was provided on behavior wherein activities or exercises were recorded on performance metrics through a digital application and discussed by the physiotherapist during follow-up [33,42,56,57]. Among the included studies, 12 involved self-monitoring for managing exercise reminders and records, viewing progress charts, and setting or modifying exercise objectives [31,32,36,39,42,44,47,55,57-59,62]. Moreover, 13 studies offered participants a wearable device with additional features, such as the ability to monitor activity intensity and visualize activity performance over time [11,33-35,37,39,42,47,56-58,61,62]. These features enabled individuals to monitor progress and receive real-time feedback on objective achievement.

A total of 18 studies applied shaping knowledge. They primarily incorporated videos or lectures on osteoarthritis (OA), the effects of PA, self-management, and coping strategies [21,32,42-44,46-50,52-54,56,59,61]. Three of these studies presented information about antecedents via multiple online physical therapy consultations using video phone services [32,36,38].

A limited subset of digital applications employed social support. The programs offered online platforms where individuals could engage in discussions pertaining to joint pain. Four studies documented the beneficial effects of engaging with social organizational structures on the rehabilitation of individuals with KOA [33,43,59,60].

The concept of natural consequences was addressed in 11 investigations [32,33,35,36,39,41-43,50,51,63], and it included information regarding health consequences, monitoring of emotional consequences, and anticipated misgivings. At each online meeting, the interventionist provided the patient with information about the benefits and costs of engaging in or refraining from a particular course of action. In addition, reminders regarding obstacles and facilitators were provided beforehand.

Comparison of behavior was addressed in 16 studies [32,33,36,42-44,49,53,56,58-63]. It was primarily implemented with behavior evidence, social comparisons, and information about the approbation of others. Three studies on group therapy prompted patients to establish a “buddy” system to change their behavior [33,56,59]. In some cases, a physiotherapist was included to provide the patient with assistance or instrumental social support. Twelve studies referred to information about other people’s perceptions of a person’s behavior and whether others would approve or disapprove of any proposed behavioral change to encourage people to decide to set overall goals [32,36,42-44,49,53,58,59,61-63]. For instance, making behavioral decisions was practiced more the following week, along with identifying obstacles to executing the behavior and devising strategies to overcome them.

A total of 16 studies referred to associations, particularly prompts, as reminders for the patient to perform a particular behavior [11,21,22,31,34,37,40,43,45,48,50,54,55,59,60]. The defined frequency, intensity, or duration of the specified behavior, along with a description of at least one context, location, time, and manner, was included. Of those, 12 involved primary distribution by the physiotherapist via short messages or email timed reminders. In addition, 8 studies involved prompts by the application’s included features [21,22,31,40,48,54,55,59].

Repetition and substitution, which involve behavioral practice or rehearsal, generalization of target behaviors, and grading tasks, were the most common components of the applications. In 27 studies, patients were required to rehearse and repeat KOA exercises [11,21,34,35,37,39-43,46,48,49,51,56,58,59,61-63]. Additionally, 1 study described neuromuscular exercises designed to enhance the physical function of the lower extremities, and the targeted behaviors were broken down into daily video bundles sent to patients [59]. Five studies divided the exercises into varying intensities and progressively increased the difficulty until the desired behaviors were achieved [31,33,45,49,63]. Individual progress and the patient’s perception of the capacity to exercise without aggravating discomfort were taken into account.

Rewards and threats were mentioned in 2 studies [32,36]. Mobile health apps were supplemented with motivation-enhancing techniques, such as praise, encouragement, and material rewards, for the achievement of specific goals.

The primary goal of regulation was to reduce negative emotions in patients. Seven studies trained users to recognize negative thoughts and reactions to them by relaxing mood through thoughts, emotions, and behaviors that affect pain [33,38,42,43,50,52,59]. One study adhered to the practice by revisiting pleasant imagery and distractions from the previous week [32]. In addition, 1 study discussed the potential for novel or alternative pain medications in applied implicit learning [43].

#### CBT

Complementary and alternative medicine therapy refers to a deliberate, intentional, and organized form of psychotherapy intervention aimed at improving psychological issues by impacting the beliefs and behaviors of patients [67,68]. CBT combines techniques to develop more adaptive cognitions and behaviors, such as psychoeducation, cognitive restructuring, relaxation therapy, and guided imagery (eg, to reduce muscle tension and autonomic arousal), as well as positive thinking training, problem-solving, and stress management [69,70].

Specifically, CBT focuses on reducing pain and distress by altering bodily sensations, catastrophic and contemplative thinking, and maladaptive behaviors, as well as enhancing self-efficacy [71,72]. Four studies addressed common CBT topics, such as catastrophizing, positive coping methods, and anxiety avoidance, through educational interactive modules and internet courses pertaining to behavior change [42,50,52,61]. The remaining 4 studies addressed common barriers to exercise (eg, pain, low confidence, weather, and relapse) and ways to overcome them (eg, increasing confidence through exercise, seeking social support, teaching proper exercise routines and postures, and promoting positive reasoning) in conjunction with programmatic elements of social cognitive theory and goal-setting strategies for exercise behaviors [32,36,43,44].

### Interactive Device Function

#### Digital Presentation Modalities

The emergence of the internet in the health field has drastically altered the medical information available to patients and the manner in which physicians and patients communicate [73]. A significant number of digital applications involving KOA utilize information and communication technology (ICT) to facilitate behavioral therapy. The included studies covered 5 types of digitization (Table 4): (1) app, (2) website, (3) teleconferencing software/remote phone contact, (4) wearable electronic device, and (5) SMS text message/telephone/email.

**Table 4:** Forms of digital applications.

Digital application type	References
App/website + SMS text message/telephone/email	[11,21,31,34,36,44,48,50,52-54,59]
App/website	[32,33,37,41,46,49,51,63]
Teleconferencing software/remote messaging	[22,40,43,45,55,60]
Wearable electronic device + SMS text message/telephone/email + app/website	[35,38,39,42,47,56,57,61]
Teleconferencing software + wearable electronic device + app/website	[58,62]

Twelve studies adopted the combination of app/website plus SMS text message/telephone/email. Physiotherapists in 2 studies provided verbal and written education or information about OA, benefits of PA or exercise, and strategies to increase adherence [11,44]. In addition, a progressive individualized home exercise program based on scientific evidence was devised, which included several lower extremity exercises and was accessible through an app or website. In 2 studies, weekly emails containing OA-specific content and resources were sent directly to patients. The emails included (1) nutritional advice; (2) an instructional video on exercise; and (3) a video on positive thinking and advice on self-care, motivation, and stress management [36,59]. Nine studies supported general health and wellness behavior change through free website support [11,21,31,34,36,48,53,54,59]. To increase patient compliance, physiotherapists conducted regular telephone counseling sessions to determine if the use of optional sessions should be based on participant preference, confidence, and success in achieving the desired behavior change. In 3 other investigations, the aforementioned functions were implemented in their entirety within a single application [44,50,52].

Eight studies used apps or websites for self-management and coping with arthritis pain through exercise [32,33,37,41,46,49,51,63]. The websites included information on PA or exercise, goal setting, action plans, pacing, medication management, diet, home exercise, understanding pain, pain management, and relaxation modules. One study explained how individuals can input data and view graphical feedback regarding the amount of exercise they have performed, their activity levels, and their mood [33].

Five investigations [22,40,43,45,60] applied teleconferencing software or telemessaging, and 3 programs provided patients with complimentary access to online webinars featuring “expert advice” [43,45,60]. Participants could ask the facilitator queries about nutrition, exercise, or positive thinking. Registered dietitians, registered psychologists, and kinesiologists led the sessions in pairs according to a rotating schedule. Two other studies addressed recommendations for the development of health behavior interventions utilizing only SMS text messaging on mobile phones [22,40].

Eight studies used the combination of wearable electronic devices plus SMS text message/telephone/email plus app/website to guide participants in setting specific, measurable, achievable, pertinent, and time-bound PA goals [35,38,39,42,47,56,57,61]. Self-monitoring is typically assessed using commercially available wrist-worn wearable activity trackers (such as Fitbit) or other similar devices. These devices collect measures and communicate them through Bluetooth to a smartphone, tablet, or computer application. Subsequently, the application transmits the data to the Fitbit server. Individuals who possess apprehensions over engaging in PA have the option to communicate their concerns via electronic mail to their physiotherapists.

Two studies [58,62] employed teleconferencing plus electronic devices plus apps. The program comprised 3 elements: (1) a 1-time knee boot camp where participants worked at home on their exercise therapy and PA goals; (2) weekly personalized in-home exercise therapy, PA, and tracking where participants received a Fitbit Inspire activity tracker; and (3) weekly physiotherapist-guided exercise therapy and activity action plans via videoconference on Zoom, optional group exercise classes, and exercise therapy and PA goal setting. In the TeleHab app, exercise therapy objective completion levels, target rating of perceived effort, and any associated pain were recorded, and Fitbit data were synchronized with the Fitbit online dashboard.

### Design Methodology

Although researchers and developers typically validate their digital applications with end users, it is uncommon for relevant studies to include end-user participation in the design phase. Of the 36 studies in our review, 8 (22%) reported end-user participation in the design phase [31,33,37,40,41,48,50,56], and 6 (75%) of these 8 studies also reported the participation of stakeholders other than end users [33,37,40,41,50,56]. These stakeholders also included caregivers, coaches, physiotherapists, and other individuals who provide care or services to the target population.

Heuristic assessments were used in a study to explore usability of the intervention for patients with KOA. Based on the outcomes of the interviews and heuristic evaluations, the program’s time structure was modified to be more flexible. In the most recent iteration, users had the option of repeating modules and adjusting module difficulty. The strategy also addressed improper website design and placement of multiple icons [31]. In addition, a study involved 3 patients with KOA who provided feedback on the prototype to inform the final design. Regarding how participants perceived interventions used outside the study setting, the majority suggested that health professionals, particularly general practitioners or physiotherapists, could deliver interventions to enhance or improve care [48].

Additional stakeholders were included in the qualitative study analysis. One study referred to experimental websites where patients with KOA and physiotherapists provided feedback on prototypes to inform the final design [40,41,50]. In a separate study, the app was also developed in collaboration with physiotherapists, physicians, and patient representatives. Named members of the project team submitted a list of 30 SMART (Specific, Measurable, Achievable, Relevant, and Time-bound) objectives related to OA treatment [41].

### Study Outcomes

A total of 23 experimental studies, 7 experimental protocols, and 6 qualitative studies were included in this literature review. The primary outcomes of using digital behavioral therapy for rehabilitation of patients with KOA were (1) pain, (2) symptoms, (3) physical functioning, and (4) PA. Moreover, the secondary outcomes included (1) self-efficacy, (2) usability and user needs, (3) health-related quality of life, (4) satisfaction, (5) negative emotions, (6) quality of sleep, (7) adherence, (8) surgical intent, and (9) understanding of the condition (Table 5).

**Table 5:** Rehabilitation outcomes for patients with knee osteoarthritis.

Outcome of the intervention	References
Pain	[11,21,22,31,32,34,36,38,41,42,45,46,49-56,59-61,63]
Symptoms	[31,35,36,41,44,47,49-52,60]
Physical function	[11,21,22,31,32,34,36,38,39,41,44-46,49,50,52-55,59,63]
Physical activity	[21,22,31,34,35,39,41,44,46,47,49-51,53-56,58,60,61]
Self-efficacy	[11,21,22,32,36,46,52,55,58-60]
Usability and user requirements	[33,37,40,42,48,49,54,56,58,62]
Satisfaction	[21,42,45,46,55,59,61,63]
Health-related quality of life	[11,21,22,34,36,46,48-51,53-55,59,63]
Negative emotions	[22,32,42,44,52,53,61]
Quality of sleep	[42,61]
Adherence	[22,36,42,43,49,55,58]
Surgical intention	[38,55,59]
Understanding of the condition	[38,51]

A total of 24 studies that aimed to reduce pain in patients with KOA were ultimately identified in this review [11,21,22,31,32,34,36,38,41,42,45,46,49-56,59-61,63]. Moreover, 12 studies reported statistically significant decreases in pain [11,21,31,32,36,38,41,42,49,59-61]. Of the 12 studies that did not demonstrate improvement, 6 involved randomized controlled trial protocols and 2 involved qualitative studies. In the studies that showed improvement, intervention durations ranged from 9 weeks to 9 months, and in those that did not show positive results, intervention durations ranged from 8 weeks to 6 months.

Overall, 21 studies assessed physical dysfunction [11,21,22,31,32,34,36,38,39,41,44-46,49,50,52-55,59,63], and of these, 6 reported statistically significant improvements [21,34,41,44,45,59]. Among the 15 studies that did not demonstrate improvement, 7 were randomized controlled trial protocols and 2 involved qualitative research. In studies that demonstrated improvement, intervention durations ranged from 3 weeks to 9 months, while in studies that demonstrated no improvement, intervention durations ranged from 4 weeks to 4 months.

A total of 12 studies measured PA outcomes [21,22,31,34,35,39,41,44,46,47,49-51,53-56,58], and 5 of them reported statistically significant improvements [34,41,47,49,60]. Among the 15 studies that did not demonstrate improvement, 6 were randomized controlled trial protocols and 2 involved qualitative research. In studies that demonstrated improvement, intervention durations ranged from 3 weeks to 9 months, while in studies that demonstrated no improvement, intervention durations ranged from 4 weeks to 12 months.

Physical symptoms were assessed in 11 studies [31,35,36,41,44,47,49-52,60], and of these, 4 studies reported statistically significant improvements [31,35,44,49]. In studies that demonstrated improvement, the intervention duration was 2 months, while in studies that demonstrated no improvement, intervention durations ranged from 2 to 9 months.

Self-efficacy was examined in 11 studies [11,21,22,32,36,46,52,55,58-60], and of these, 3 studies reported statistically significant improvements [11,36,58]. A total of 15 studies reported health-related quality of life [11,21,22,34,36,46,48-51,53-55,59,63], and of these, 3 studies reported statistically significant improvements [11,49,59]. Satisfaction was assessed in 8 studies [21,42,45,46,55,59,61,63], and of these, 1 study reported significant improvements [61]. Seven studies reported improvements in self-reported negative affect after the intervention [22,32,42,44,52,53,61]. Adherence was evaluated in 7 studies [22,36,42,43,49,55,58], and of these, 2 studies reported statistically significant improvements [22,58]. Sleep quality was assessed in 2 studies [42,61], and of these, 1 study [61] reported significant improvements. Three studies examined surgical intent [38,55,59]; however, none of the studies reported significant changes in patients’ surgical intent. Patients’ understanding of their condition was examined in 2 studies [38,51]; however, no improvement was identified.

### Application Deficiencies

The design recommendations of users and stakeholders for the app were reported at different stages of the final app study design. Two studies identified the negative emotions associated with the app [31,56]. For instance, Östlind et al [56] found that a wearable activity tracker facilitated PA in various ways and increased the awareness of the optimal number of steps to treat OA symptoms. However, not all participants found the wearable activity tracker to be motivating, and in some cases, if they missed a weekly PA, the app’s prompts about PA caused them to feel anxious and frustrated [31].

Two studies examined the efficacy of applying various characteristics. For instance, the study by Lindberg et al [50] demonstrated the combined efficacy of education, exercise therapy, and internet-based CBT, but was unable to distinguish between these interventions individually. Moreover, Dunphy et al [49] suggested that some participants viewed the physiotherapist’s participation as positive, customizing the digital program and monitoring their progress. Others described it as restrictive, particularly if the physiotherapist did not understand how the digital program operated.

In terms of how to promote PA, the studies suggested various potential solutions. For instance, setting and achieving a daily step objective would motivate individuals to walk more than usual [56]. In addition, there was a strong desire for support from health care professionals who could monitor and guide progress; reinforce health messages; and offer reassurance, motivation, and encouragement. In the interviews, peer support through online communities (eg, forums and blogs) was also mentioned as a positive feature where people could share their experiences and learn from and support others experiencing joint pain, although users felt that it needed to be supervised to prevent inaccurate and inappropriate posts [37].

## Discussion

### Overview

This review presents an analysis of 36 papers that examined the use of digital behavioral therapies for patients with KOA. The analysis provides insights into 3 key areas: (1) the effectiveness of digital and behavioral therapies, (2) the role of digital technologies in these treatments, and (3) the importance of involving users and stakeholders throughout the design phase.

### Digitalization and Behavioral Therapy

Several digital applications designed to provide behavioral therapy–based approaches for KOA were reviewed. Various theories of behavior modification and cognition were incorporated into these applications to increase their applicability and efficacy. In this review, we discuss behavioral therapy elements that are frequently combined to form applications.

Physiotherapists or applications, goal setting based on the user’s current physical status, and BCTs derived from a control theory framework can enhance long-term sustained exercise in patients with KOA [74]. Positive reinforcement of progressive PA is the most essential element of goals and planning [75]. Gradual increases in PA alter the perception that PA is associated with pain and increase confidence in improving PA performance, resulting in favorable physical (eg, physical ability, muscle strength, and joint flexibility) and psychological (eg, self-esteem, pain perception, and anxiety) changes. Repetition and substitution are intended to boost application engagement and health-related behaviors. A “severe” to “extremely severe” program and specific dosages are designed to stimulate strength gains, resulting in enhanced function. Physiotherapists assisted participants in devising a PA program intended to increase PA, with exercises targeting the hips, knees, and ankles, including sit-to-stand exercises and seated knee extensions. Our findings indicate that the aforementioned 2 BCTs were covered in virtually all of the applications we reviewed.

It has been demonstrated that reminders increase adherence to unsupervised home strengthening exercises [76]. This feature provides personalized behavior change messages to help patients with KOA surmount barriers to exercise participation. The physiotherapist or the application sends reminder messages based on completion to assist and remind the user to reach their exercise objectives. In the mobile app or wearable device, graphical displays were used to monitor workout adherence and provide feedback. These approaches provided the ability to register the completion of weekly workout sessions and send regular messages to encourage weekly workout participation. Despite the prevalence of wearable devices, their effectiveness in enhancing PA has been questioned. These devices frequently incorporate motivational techniques, such as self-monitoring and real-time feedback, but rarely address skills such as action planning and problem solving, which are essential for altering PA behaviors. Some studies also examined common CBT topics, such as catastrophizing, positive coping strategies, and fear avoidance [42]. Specifically, supervised exercise therapy through patient education enables users to access information regarding OA treatment modalities as well as topics such as the advantages of a healthy lifestyle, PA, vitality, and nutrition.

Our analysis revealed that OA digital management applications may be an alternative to traditional therapy and may further assist with the implementation of OA standards in the wider community [77,78]. However, the engagement of a physiotherapist is a vital aspect. Most participants had favorable experiences with their assigned physiotherapist and were motivated by the daily contact and the support and encouragement provided [79].

### Insights From Technology

The use of digital technologies in communication by the patient care team can contribute to the enhancement of information flow, facilitation of patient information retention, improvement of information accessibility and portability, customization of information based on individual needs, and provision of tools for patients to actively participate in their health care [75,80]. In our analysis, the process of digitization manifested through a convergence of several technological mediums, including apps and websites, text messaging, phone calls, emails, and wearable gadgets.

New patient self-management programs have emerged in recent years, demonstrating the importance and efficacy of eHealth interventions such as websites and mobile apps. Current topics include PA, exercise, goal setting, action plans, pacing, medication management, nutrition, at-home exercise, pain comprehension, pain management, and relaxation. They may be combined with multimedia technologies to facilitate the sharing of content and with human-computer interface technologies to enhance accessibility.

Most health and medical apps fail to retain users beyond 90 days. This is due to their missing potential for facilitating disease management and provider-patient communication. The development of teleconferencing software or telematic messaging technology is supported by reminders and distinct objectives [81]. The development of text messaging program functionality and message libraries (including message types, message frequency, and program interaction levels) has the potential to increase adherence to unsupervised home exercise.

Wearable electronic devices aid in tracking the user’s daily activities and offer continuous visualization support. However, such devices typically carry a greater risk of privacy invasion and social stigmatization. Our findings indicate that none of the studies specifically addressed privacy concerns. Therefore, designers of pertinent systems should be encouraged to consider these factors more thoroughly. In addition, current wearable electronic devices collect limited data, and their primary function is still to provide feedback on physiological signals (steps, consumption, asymmetry, etc), lacking the accumulation of electrical signals unique to KOA. In the future, machine learning can be introduced into ubiquitous devices to predict patient activity in order to improve patient care.

In the reviewed studies, the combination of app/website plus SMS text message/telephone/email was the most common. It has been shown that text messaging programs combined with unsupervised web-based exercise can reduce pain and dysfunction in patients with KOA [22,40]. Although the addition of wearable electronic devices would improve the intervention process by providing more accurate monitoring data, the experience would not be enhanced. However, the cost of ubiquitous electronic devices and the complexity of their operation continue to prevent their widespread adoption.

### Insights From the User or Stakeholder Experience

Less than half of the reviewed studies reported structured user or stakeholder participation in the design phase of their systems, according to our analysis. Nonetheless, there is a distinct trend indicating that an increasing number of studies are emphasizing the significance of involving users in the design and development phases (and not just the validation or deployment phases). Specifically, we summarize a set of emerging user or stakeholder involvement design trends in emerging research on the application of digital behavioral therapy for KOA. Based on the interviews conducted for the qualitative study, issues were identified with the digital app user experience of KOA patients [52,56,62].

First, direct patient feedback was not taken into account when developing the content of the apps, and the content was based solely on the knowledge and experience of hospital staff. In future studies, patients should play a more important role in the development of the content of patient-specific apps, evaluating and further optimizing the preferable mode of information for outcomes [26]. Moreover, there is a need for a structured collaborative design involving patients, physicians, and researchers in order to establish multiple collaborative processes based on shared concepts, mutual learning, and respect for diversity and divergent opinions [82].

Second, digital support is regarded as an integral component of OA care. An ideal approach would be one that combines traditional OA care with digital OA care to offer solutions for personalized, comprehensive, straightforward, dependable, and continuous hybrid care [56,81]. The experience of digital applications is divided into 4 subcategories [79]: (1) simplicity of implementation, (2) flexibility in choosing time and location, (3) significance of interaction with health care professionals, and (4) additional motivating factors. Consequently, our research contends that digital applications must incorporate and differentiate between various user experience stages.

The functional requirements of apps that patients, physicians, and researchers deemed most essential, convenient, desirable, and actionable differed significantly. Participants in the studies agreed, despite their differences, that minimum viable products should be electronic, should monitor patients’ symptoms and activities, and should include features tailored to factors identified by patients and physicians as well as self-management strategies based on international guidelines. Over the course of the study, participants came to a consensus regarding the order of their functional requirements. Visual symptom mapping, goal setting, exercise programs, daily monitoring, and self-management strategies had the highest priorities.

### Future Work

After conducting a thorough study, a set of recommendations were summarized to improve the quality of applications. These recommendations are focused on making the process more efficient and improving future design and development endeavors. Suggestions for future studies are presented below.

#### Digital Applications that Provide Comprehensive Monitoring of the Entire Treatment Process

To enhance the rehabilitation of patients, it is necessary to thoroughly investigate the efficacy and impact of different behavioral therapies. This will enable the development of more precise application strategies. Multiple behavioral therapies should be integrated to cover all aspects of the rehabilitation process, including rehabilitation exercises, status monitoring, and self-screening, in order to ensure that patients receive comprehensive care. Simultaneously, by promptly understanding the wants of patients, we can bolster their trust in the process of recuperation. This customized service model can enhance patient happiness and confidence, while also facilitating the seamless advancement of the rehabilitation process. Further investigation can enhance our comprehension by examining particular facets of digital health care platforms, such as network effects and the strategic management of platform ecosystem innovation [83].

#### Data Analysis Requirements of Patients Based on Intelligent Algorithms

Artificial intelligence algorithms are crucial for extracting important insights from vast quantities of data and offering therapists precise recommendations. As an illustration, the algorithm may examine a patient’s exercise data while they are undergoing rehabilitation and detect tiny modifications. This helps the therapist in fine-tuning the intensity and substance of the training. Furthermore, through the comparison of data from many patients, the algorithm can investigate the efficacy of different treatments for specific patient groups, thereby establishing a strategic foundation for later treatment decisions. Significantly, these data serve the dual purposes of enhancing the precision and pertinence of treatment and evaluating the impact of different behavioral interventions. In conventional rehabilitation programs, this procedure is frequently based on subjective criteria. By employing a data-driven methodology, we can impartially evaluate the true efficacy of each technique, establishing a strong basis for further research and applications.

#### Community-Based Rehabilitation Scenarios With Digital Technology Integration

In order to enhance the adoption of digital behavioral therapy in rehabilitation, our objective is to extend its implementation from the domestic setting to the broader social community rehabilitation setting. The community rehabilitation setting offers a platform for patients to engage and assist one another, and fosters patients’ motivation and assurance in their recovery process. For instance, an internet-based community can be established to facilitate patients in exchanging their recovery experiences, offering reciprocal motivation and assistance, and deliberating the obstacles and remedies related to the recovery journey. Additionally, offline activities, including rehabilitation lectures, group seminars, and interactive games, can be arranged to augment patients’ social interaction and foster a sense of team camaraderie. Strong partnerships can be fostered with medical organizations and rehabilitation professionals in the community to offer comprehensive assistance for patient recovery. This not only enhances patient rehabilitation outcomes and quality of life, but also fosters the integration and optimization of community rehabilitation resources and promotes overall progress in the field of patient recovery.

### Limitations

This research has some limitations. Initially, our evaluation thoroughly examined the present state of digital behavioral therapy applications using qualitative analysis. However, it is indisputable that quantitative analysis yields more robust clinical value. Hence, a complete quantitative meta-analysis will be conducted to assess the efficacy of digital behavioral therapy applications. Future research will integrate both qualitative and quantitative analyses to provide a more thorough evaluation. In addition, our search was limited to English-language research publications, and it is possible that there are significant findings in other languages. The search for research papers was restricted to 5 databases (Web of Science, ScienceDirect, PubMed, Ovid, and Embase). As algorithms are added to digital intervention tools, additional computer science databases, such as IEEE and ACM, could be added to increase the comprehensiveness of the assessment. Furthermore, this review was limited by the search criteria employed and the time period during which the papers were published. However, a focus on the last 10 years went a long way in ensuring that this systematic review includes the most recent research.

### Conclusion

This review provides an overview of digital behavioral therapy applications for patients with KOA. In this systematic review, 36 studies were examined. The results demonstrate 14 BCTs and show that behavioral cognitive therapies are frequently combined when developing digital applications. The most prevalent areas were “goals and planning” and “repetition and substitution,” which were frequently used to develop PA goals and PA behavior adherence. The most prevalent combination strategy was app/website plus SMS text message/telephone/email, which has tremendous potential. Consequently, this research provided results for digital applications in terms of pain relief, physical function improvement, self-confidence, and improvement in health-related quality of life among patients with KOA. Nevertheless, there was a shortage of evidence indicating enhanced surgical intention, compliance, and disease knowledge. Subsequent quantitative analysis of this phenomenon is required in the future. Moreover, the incorporation of several stakeholders in the design and development stages might enhance the user experience, considering the distinct variations in their requirements. Based on the findings, digital applications should incorporate various stages of user experience and should include a combination of traditional and digital solutions for OA care.

## Appendix

Multimedia Appendix 1 PRISMA (Preferred Reporting Items for Systematic Reviews and Meta-Analyses) checklist.

Multimedia Appendix 2 Mixed Methods Appraisal Tool (MMAT), version 2018.

Multimedia Appendix 3 The literature search results.

Multimedia Appendix 4 Methodological quality assessment using the Mixed Methods Appraisal Tool (MMAT).

## Abbreviations

BCT: behavioral change technique
CBT: cognitive behavioral therapy
KOA: knee osteoarthritis
MeSH: Medical Subject Headings
MMAT: Mixed Methods Assessment Tool
OA: osteoarthritis
PA: physical activity
