# Supplementary material for: Rehabilitation Applications Based on Behavioral Therapy for People With Knee Osteoarthritis: Systematic Review
Source: JMIR Mhealth Uhealth. 2024 May 2;12:e53798. doi: 10.2196/53798 (PMC11099817; doi:10.2196/53798)
Supplement: Multimedia Appendix 1 [file mhealth_v12i1e53798_app1.pdf]

PRISMA 2020 Checklist

Section and Topic	Item #	Checklist item	Location where item is reported
**TITLE**			
Title	1	Rehabilitation Application based on Behavioral Therapy for People With Knee Osteoarthritis: Systematic Review	
**ABSTRACT**			
Abstract	2	Background: The development of digital applications of behavioral therapies to support patients with knee osteoarthritis (KOA) has attracted increasing attention in the field of rehabilitation. This paper presents a systematic review of research on the digital application of behavioral therapies for people with KOA.<br>Objective: The main study aims to describe the characteristics of relevant digital applications, with a special focus on the current state of behavioral therapy, digital interaction technologies, and user participation in design. The secondary aim is to summarize intervention outcomes and user evaluations of digital applications.<br>Methods: A systematic literature search was conducted using the keywords "Knee Osteoarthritis", "Behavior Therapy" and "Digitization" within the following databases (from 2013 to July 2023): Web of Science, EMBASE, Science Direct, Ovid and PubMed. A total of 2 researchers independently screened and extracted the data.<br>Results: Results 36 studies met the inclusion criteria and were further analyzed. Behavioral change techniques (BCT) and behavioral cognitive therapy (CBT) are frequently combined when developing digital applications. The most prevalent are goals and planning, as well as repetition and replacement, which are frequently used to develop physical activity (PA) goals and adherence. The most prevalent combination strategy employs apps/websites plus SMS/phone/email, which demonstrates tremendous potential. Additionally, when designing the application to explore the research area in depth, the requirements of each stakeholder must be considered.<br>Conclusion: The results demonstrate that setting goals and planning, repetition and replacement are frequently used to develop PA goals and PA behavior adherence. The most prevalent combination strategy employs apps/websites plus SMS/phone/email, which demonstrates tremendous potential. Moreover, incorporating several stakeholders in the design and development stages might enhance the user experience, considering the distinct variations in their requirements.	
**INTRODUCTION**			
Rationale	3	Currently, there are evaluations investigating the rehabilitative impacts of digitalization in KOA and highlighting the significance of behavioral theory in some applications [19]. Nevertheless, there is a shortage of thorough exposition of the behavioral theory in digital applications, as well as an absence of study about the suitability of these applications from the patient's point of view. Thus, this study offers a methodical and thorough examination of digital applications rooted in behavioral therapy. It shifts the focus of digital applications from mere practical usability to providing support for behavioral change theories. Additionally, it meticulously analyzes the functional reasoning behind various products, thereby serving as a comprehensive guide for designing future digital interventions.	
Objectives	4	Hence, the objective of this review is: (1) to provide a concise overview of the existing landscape of digital behavioral therapy applications for individuals diagnosed with KOA and examine the potential of patient digital applications in augmenting the rehabilitation process for KOA patients; (2) to present a comprehensive analysis of the underlying psychological theories, fundamental mechanisms, design methodologies, typical attributes, efficacy of treatment outcomes, and patient preferences pertaining to this particular mode of recovery intervention.	
**METHODS**			
Eligibility criteria	5	On this basis, we anticipated that this study would: (1) generalize and summarize the digital applications used in behavioral therapy; and (2) describe the overall research status and research trends of these digital applications.<br>**Inclusion Criteria**<br>Adult participants (≥18 years old) with KOA diagnosed by self-reported symptoms or imaging<br>Patients have access to digital applications<br>Any form of intervention/treatment based on the inclusion of at least one behavioral treatment is delivered through any digital application (e.g., website or app) within any time frame.<br>The described interventions are compared to waiting list control (no intervention) or alternative (standard) delivery modalities (e.g., face-to-face,	

PRISMA 2020 Checklist

<table>
<tr><th>Section and Topic</th><th>Item #</th><th>Checklist item</th><th>Location where item is reported</th></tr>
<tr><td></td><td></td><td>classroom-based, and printed materials/handouts), non-digital self-management interventions, and non-interactive digital interventions (e.g., web pages with flat copies).
Exclusion Criteria
KOA patients not included;
Non-digital interventions;
Does not include behavioral therapy theories;
Research protocols, reviews, conceptual articles, case studies/discussion papers, conference abstracts
Market research;
Digitization is not designed for the recovery process;
Articles not written in English;
Duplicate reports of the same study from different sources.</td><td></td></tr>
<tr><td>Information sources</td><td>6</td><td>Literature searches were conducted in five databases: Web of Science, EMBASE, Science Direct, Ovid and PubMed. The selection of these databases was based on their provision of comprehensive access to full-text journals and conference proceedings pertaining to prominent conferences and meetings focused on digital technology and medicine. Similar search strategies apply to the remaining three databases. Relevant articles published between January 2013 and July 2023 were gathered. The articles included refereed journal papers and peer-reviewed conference proceedings articles. Only articles written in English were included.</td><td></td></tr>
<tr><td>Search strategy</td><td>7</td><td><table>
<tr><td>Mesh</td><td>Boolean Logic—Search Strings</td></tr>
<tr><td>Knee Osteoarthritis</td><td>“Knee Osteoarthritides” OR “Knee Osteoarthritis” OR “Osteoarthritis of Knee” OR “Osteoarthritis of the Knee”</td></tr>
<tr><td>Behavior Therapy</td><td>“Behavior Therapies” OR “Behavior Treatment” OR “Conditioning Therapy” OR “Conditioning Therapies” OR “Behavior Change Techniques” OR “Behavior Change Technique” OR “Behavior Modification” OR “Behavior Modifications” OR “Dialectical Behavior Therapies” OR “Cognitive Behavioral Therapies” OR “Cognitive Therapy” OR “Cognitive Behavior Therapy” OR “Cognitive Psychotherapy” OR “Cognition Therapy” OR “Cognitive Behavior Therapies” OR “Cognitive Behavior Therapy”</td></tr>
<tr><td>Digitization</td><td>“Telemedicine” OR “Mobile Health” OR “Telehealth” OR “ehealth” OR “mhealth” OR “Email” OR “E—mail” OR “Mobile” OR “Smartphone” OR “smart—phone” OR “smart telephone” OR “Tablet” OR “cell” OR “hand—held” OR “Cell Phone” OR “handheld” OR “Remote Consultation” OR “Teleradiology” OR “Telenursing” OR “Computer Systems” OR “Computer—Assisted Instruction” OR “Internet” OR “web” OR “computer” OR “Digital Technologies” OR “APP” OR “Social Media” OR “Internet—Based Intervention” OR “Mobile Application” OR “Mobile App” OR “Smartphone App” OR “Portable Software Application”</td></tr>
</table></td><td></td></tr>
<tr><td>Selection process</td><td>8</td><td>Figure 1 provides a summary of the outcomes at various phases of article selection.</td><td></td></tr>
<tr><td>Data collection process</td><td>9</td><td>The authors (DZ and JZ) were assisted in the literature search by an experienced librarian well versed in medical database searching. The review was conducted in accordance with the Preferred Reporting Items for Systematic Reviews and Meta-Analyses (PRISMA) guidelines.</td><td></td></tr>
<tr><td>Data items</td><td>10a</td><td>The dataset comprised study information, participant characteristics, type of psychotherapy, type of digital tool, study duration, and outcome measures, including pain and function scores.</td><td></td></tr>
</table>

PRISMA 2020 Checklist

Section and Topic	Item #	Checklist item	Location where item is reported
	10b	Application deficiencies	
Study risk of bias assessment	11	The Mixed Methods Assessment Tool (MMAT) was used to evaluate the methodological quality of the studies included. It was initially created in 2006 through a comprehensive analysis of systematic evaluations that integrated qualitative and quantitative evidence. In 2018, a revised version of the MMAT was developed by assessing its usefulness, reviewing key assessment tools in the literature, and conducting a modified e-Delphi study involving methodology experts to determine the essential criteria. The MMAT evaluates the caliber of research employing qualitative, quantitative, and mixed approaches. The primary emphasis is on methodological standards, which encompass five fundamental quality criteria for five distinct study designs: (a) qualitative, (b) randomized controlled, (c) non-randomized, (d) quantitative descriptive, and (e) mixed methods.	
Effect measures	12	This is a qualitative study	
Synthesis methods	13a	This is a qualitative study	
	13b		
	13c		
	13d		
	13e		
	13f		
Reporting bias assessment	14	Supplementary table 1	
Certainty assessment	15	This is a qualitative study	
**RESULTS**			
Study selection	16a	Based on the search strategy, 2,975 articles were found initially. 2,507 articles remained after title and abstract screening and removal of duplicates. Next, full-text articles were chosen based on inclusion and exclusion criteria. Included were a total of 131 articles explicitly related to digital behavioral therapy for KOA. 4 articles were chosen following a manual search of the references for articles that were cited. Final consideration was given to 36 articles for systematic evaluation.	
	16b	4 additional records were found through cross-referencing of bibliographies and the other by contacting the corresponding author(s).	
Study characteristics	17	Results are listed in Table 2	
Risk of bias in studies	18	12 studies achieved 100% of the quality assessment criteria (Supplementary table 2). 15 studies (fulfilled 60%-80% of the quality assessment criteria. 2 studies met 40% of the quality assessment criteria. The remaining 7 studies could not be evaluated for their quality due to the absence of results. Nevertheless, the application description portion that is involved in the studies remains highly valuable for analysis.	
Results of individual studies	19	Results are listed in Table 2	
Results of syntheses	20a	Digital approach to behavioral therapy<br>The majority of digital applications for KOA rehabilitation are based on BCT. BCT (achieving objectives, setting goals, restructuring beliefs, and inducing acceptance) is applicable to addressing the central issues of initiating and maintaining PA. The primary categories of BCT used in the reviewed studies were based on Michie and colleagues' V1 Taxonomy of Behavior Change, which was devised by behavior change researchers. The taxonomy comprises 93 distinct BCTs organized into 16 hierarchical structures and has been extensively used in the literature on behavior	

Section and Topic	Item #	Checklist item	Location where item is reported
		change (Table 3): (1) goals and planning; (2) feedback and monitoring; (3) social support; (4) shaping knowledge; (5) natural consequences; (6) behavioral comparisons; (7) associations; (8) repetition and substitution; (9) outcome comparisons; (10) rewards and threats (11) regulation; (12) presuppositions; (13) identity; (14) intended consequences; (15) self-confidence; and (16) implicit learning.	
	20b	Digitization and application forms<br>Internet's emergence in the health field has drastically altered the medical information available to patients and the manner in which physicians and patients communicate. A significant number of KOA digital applications utilize information and communication technology (ICT) to facilitate behavioral therapy. The research covered five types of digitization (Table 4): (1) apps, (2) websites, (3) teleconferencing software/remote phone contact, (4) wearable electronic devices, and (5) text messages/phone calls/emails.	
	20c	Rehabilitation outcomes for patients with KOA<br>A total of 23 experimental studies, 7 experimental protocols and 6 qualitative studies were included in this literature review. The primary outcomes of using digital behavioral therapy for rehabilitation of KOA patients were (1) pain, (2) symptoms, (3) physical functioning, and (4) PA. Other secondary outcomes included (1) self-efficacy; (2) usability and user needs; (3) health-related quality of life; (4) satisfaction; (5) negative emotions; (6) quality of sleep; (7) adherence; (8) surgical intent; and (9) understanding of condition.	
	20d	Application deficiencies<br>Users' and stakeholders' design recommendations for the app were reported at different stages of the final app study design. 2 investigations identified the negative emotions associated with the app. For instance, Stlind et al found that WAT facilitated PAs in various ways and increased their awareness of the optimal number of steps to treat OA symptoms. However, not all participants found the WAT to be motivating, and in some cases, if they missed a weekly PA, the app's prompts about PA caused them to feel anxious and frustrated.	
Reporting biases	21	This is a qualitative study.	
Certainty of evidence	22	This is a qualitative study.	
**DISCUSSION**			
Discussion	23a	Digitalization and Behavioral Therapy<br>Our analysis reveals that while OA digital management applications may be an alternative to traditional therapy and further assist the implementation of OA standards in the wider community. However, the engagement of PT is a vital aspect. Since most participants had favorable experiences with their assigned PT and were motivated by the daily contact and the support and encouragement provided.	
	23b	Insights from Technology<br>In the reviewed studies, apps/websites + SMS/phone/email was the most common combination. It has been shown that text messaging programs combined with unsupervised web-based exercise reduce pain and dysfunction in patients with KOA. Although the addition of wearable electronic devices would improve the intervention process by providing more accurate monitoring data, the experience would not be enhanced. However, the cost of ubiquitous electronic devices and the complexity of their operation continue to prevent their widespread adoption.	
	23c	Insights from the user/stakeholder experience<br>The functional requirements of apps that patients, physicians, and researchers deemed most essential, convenient, desirable, and actionable differed significantly. Participants in the study agreed, despite their differences, that MVPs should be electronic, should monitor patients' symptoms and activities, and should include features tailored to factors identified by patients and physicians as well as self-management strategies based on international guidelines. Over the course of the study, participants came to a consensus regarding the order of their functional requirements. Visual symptom mapping, goal setting, exercise programs and daily monitoring, and self-management strategies were the highest priorities.	
	23d	Future development<br>Our review summarizes pertinent application design recommendations that may facilitate and improve future design and development. Meanwhile,	

PRISMA 2020 Checklist

Section and Topic	Item #	Checklist item	Location where item is reported
		for the future trajectory of research on digital behavioral therapy for KOA patients, we suggest the following: a) rational use of data to determine the recovery status by collecting patients' time-series data and providing relative responses to improve patients' adherence; b) research on the effectiveness/contribution of each behavioral therapy method to KOA recovery and develop more targeted applications; c) application of more behavioral treatments that comprehensively encompass the entire rehabilitation process (rehabilitation exercises, status monitoring, etc.); d) expanding digital behavioral therapy applications from the home to more social community-based rehabilitation settings and designing relevant intervention models; and e) future research may broaden our understanding by investigating specific aspects of digital healthcare platforms, such as network effects and platform ecosystem innovations of strategic management。	
**OTHER INFORMATION**			
Registration and protocol	24a	The present review was performed in accordance with the Preferred Reporting Items for Systematic Reviews and Meta-Analysis (i.e., "PRISMA") and guidelines published in the Cochrane Handbook of Systematic Evaluation.	
	24b	This is a qualitative study about digital application.	
	24c	CRD42023430716	
Support	25	Non-financial support	
Competing interests	26	No any competing interests of review authors.	
Availability of data, code and other materials	27	Specific data can be requested from the authors, if needed: template data collection forms; data extracted from included studies; data used for all analyses; analytic code; any other materials used in the review.	

From: Page MJ, McKenzie JE, Bossuyt PM, Boutron I, Hoffmann TC, Mulrow CD, et al. The PRISMA 2020 statement: an updated guideline for reporting systematic reviews. BMJ 2021;372:n71. doi: 10.1136/bmj.n71

For more information, visit: http://www.prisma-statement.org/
