# Supplementary material for: Rehabilitation Applications Based on Behavioral Therapy for People With Knee Osteoarthritis: Systematic Review
Source: JMIR Mhealth Uhealth. 2024 May 2;12:e53798. doi: 10.2196/53798 (PMC11099817; doi:10.2196/53798)
Supplement: Multimedia Appendix 3 [file mhealth_v12i1e53798_app3.pdf]

Multimedia Appendix 3. The literature search results.

PubMed

#	Searches	Results
1	" Knee Osteoarthritis "[Mesh]: (Knee Osteoarthritides) OR (Knee Osteoarthritis) OR (Osteoarthritis of Knee) OR (Osteoarthritis of the Knee)	51,014
2	"Behavior Therapy"[Mesh]: ((Behavior Therapies) OR (Behavior Treatment) OR (Conditioning Therapy) OR (Conditioning Therapies) OR (Behavior Change Techniques) OR (Behavior Change Technique) OR (Behavior Modification) OR (Behavior Modifications))	413,487
3	"Dialectical Behavior Therapy"[Mesh]: (Dialectical Behavior Therapies)	1,147
4	"Cognitive Behavioral Therapy"[Mesh]: ((Cognitive Behavioral Therapies) OR (Cognitive Therapy) OR (Cognitive Behaviour Therapy) OR (Cognitive Psychotherapy) OR (Cognition Therapy) OR (Cognitive Behavior Therapies) OR (Cognitive Behavior Therapy))	167,761
5	2 OR 3 OR 4	521,679
6	"Telemedicine"[Mesh]: (Telemedicine) OR (Mobile Health) OR (Telehealth) OR (ehealth) OR (mhealth)	121,198
7	"Electronic Mail"[Mesh]: (Email) OR (E-mail)	210,066
8	"Mobile Applications"[Mesh]: (Smartphone) OR (smart-phone) OR (smart telephone) OR (Tablet) OR (cell) OR (hand-held) OR (Cell Phone) OR (handheld) OR (Remote Consultation) OR (Teleradiology) OR (Telenursing) OR (Computer Systems) OR (Computer-Assisted Instruction) OR (computer) OR (Digital Technologies) OR (APP) OR (Social Media) OR (Internet-Based Intervention) OR (Mobile Application) OR (Mobile App) OR (Smartphone App) OR (Portable Software Application)	10,244,327
9	"Internet Use"[Mesh]: (Internet) OR (web)	333,937
10	6 OR 7 OR 8 OR 9	10,612,114
11	1 AND 5 AND 10	144
12	limit 11 to yr="2013 - 2023" and Limit 11 to English language	142

Web of Science

#	Searches	Results
1	(Knee Osteoarthritides) OR (Knee Osteoarthritis) OR (Osteoarthritis of Knee) OR (Osteoarthritis of the Knee)	94,764

2	(Behavior Therapies) OR (Behavior Treatment) OR (Conditioning Therapy) OR (Conditioning Therapies) OR (Behavior Change Techniques) OR (Behavior Change Technique) OR (Behavior Modification) OR (Behavior Modifications) OR (Dialectical Behavior Therapies) OR (Cognitive Behavioral Therapies) OR (Cognitive Therapy) OR (Cognitive Behaviour Therapy) OR (Cognitive Psychotherapy) OR (Cognition Therapy) OR (Cognitive Behavior Therapies) OR (Cognitive Behavior Therapy)	3,791,214
3	TS= (Telemedicine) OR (Mobile Health) OR (Telehealth) OR (ehealth) OR (mhealth) OR (Email) OR (E-mail) OR (Mobile) OR (Smartphone) OR (smart-phone) OR (smart telephone) OR (Tablet) OR (cell) OR (hand-held) OR (Cell Phone) OR (handheld) OR (Remote Consultation) OR (Teleradiology) OR (Telenursing) OR (Computer Systems) OR (Computer-Assisted Instruction) OR (Internet) OR (web) OR (computer) OR (Digital Technologies) OR (APP) OR (Social Media) OR (Internet-Based Intervention) OR (Mobile Application) OR (Mobile App) OR (Smartphone App) OR (Portable Software Application)	33,977,902
4	1 AND 2 AND 3	1,336
5	limit 4 to yr="2013 - 2023" and Limit 4 to English language	1,320

Ovid

#	search	Results
1	'knee osteoarthritis'/exp OR 'knee osteoarthritis'	24,058
2	(Behavior Therapies) OR (Behavior Treatment) OR (Conditioning Therapy) OR (Conditioning Therapies) OR (Behavior Change Techniques) OR (Behavior Change Technique) OR (Behavior Modification) OR (Behavior Modifications) OR (Dialectical Behavior Therapies) OR (Cognitive Behavioral Therapies) OR (Cognitive Therapy) OR (Cognitive Behaviour Therapy) OR (Cognitive Psychotherapy) OR (Cognition Therapy) OR (Cognitive Behavior Therapies) OR (Cognitive Behavior Therapy)	47,989
3	(Telemedicine) OR (Mobile Health) OR (Telehealth) OR (ehealth) OR (mhealth) OR (Email) OR (E-mail) OR (Mobile) OR (Smartphone) OR (smart-phone) OR (smart telephone) OR (Tablet) OR (cell) OR (hand-held) OR (Cell Phone) OR (handheld) OR (Remote Consultation) OR (Teleradiology) OR (Telenursing) OR (Computer Systems) OR (Computer-Assisted Instruction) OR (Internet) OR (web) OR (computer) OR (Digital Technologies) OR (APP) OR (Social Media) OR (Internet-Based Intervention) OR (Mobile Application) OR (Mobile App) OR (Smartphone App) OR (Portable Software Application)	7,109,277
4	1 AND 2 AND 3	198
5	limit 4 to yr="2013 - 2023" and Limit 4 to English language	140

EMBASE

#	Searches	Results
1	'knee osteoarthritis'/exp OR 'knee osteoarthritis'	48,987
2	'behavior therapy'/exp OR 'behavior therapy'	87,483
3	‘Behavior Treatment’:ab,ti OR ‘Conditioning Therapy’:ab,ti OR ‘Behavior Change Technique’:ab,ti OR ‘Behavior Modification’:ab,ti OR ‘Osteoarthrosis’:ab,ti	9,298
4	exp Dialectical Behavior Therapy/	1,886
5	exp Cognitive Behavioral Therapy/	39,701
6	‘Cognitive Therapy’:ab,ti OR ‘Cognitive Psychotherapy’:ab,ti OR ‘Cognitive Behavior Therapy’:ab,ti	8,784
7	2 OR 3 OR 4 OR 5 OR 6	106,475
8	Exp Telemedicine/	86,435
9	Exp Telehealth/	94,600
10	Exp E-mail/	92,211
11	Exp Smartphone/	36,748
12	Exp teleconsultation/	17,107
13	Exp ‘mobile application’/	28,059
14	Exp ‘digital health’/	15,521
15	Exp teleradiology/	2,404
16	Exp telenursing/	592
17	Exp computer system/	30,785
18	‘Internet’:ab,ti OR ‘web’:ab,ti OR ‘computer’:ab,ti OR ‘Digital Technologies’:ab,ti OR ‘Social Media’:ab,ti OR ‘Portable Software Application’:ab,ti OR ‘Mobile Health’:ab,ti OR ‘ehealth’:ab,ti OR ‘mhealth’:ab,ti OR ‘Computer-Assisted Instruction’:ab,ti	669,135
19	8 OR 9 OR 10 OR 11 OR 12 OR 13 OR 14 OR 15 OR 16 OR 17 OR 18	892,488
20	1 AND 7 AND 19	47
21	limit 20 to yr="2013 - 2023"	45
22	Limit 21 to English language	45

Science Direct

#	Searches	Results
1	(Knee Osteoarthritis) AND (Behavior Therapy) AND (Telemedicine OR Internet-Based Intervention OR Email OR “Smart phone” OR Internet OR web OR “mobile Application”)	12,200
2	limit 7 to yr="2013 - 2023"	1,334

3	Limit 2 to English language	1,328
