# Supplementary material for: Rehabilitation Applications Based on Behavioral Therapy for People With Knee Osteoarthritis: Systematic Review
Source: JMIR Mhealth Uhealth. 2024 May 2;12:e53798. doi: 10.2196/53798 (PMC11099817; doi:10.2196/53798)
Supplement: Multimedia Appendix 4 [file mhealth_v12i1e53798_app4.doc]

Supplementary table 2 Methodological quality assessment using the Mixed Methods Appraisal Tool (MMAT)

|  | Author | Screening questions | | Methodological quality criteria | | | | |  |
| --- | --- | --- | --- | --- | --- | --- | --- | --- | --- |
|  |  | S1 | S2 | 1 | 2 | 3 | 4 | 5 | All |
| [31] | D. Bossen | 1 | 1 | 1 | 1 | 1 | 0 | 1 | 4^c^ |
| [32] | C. Rini | 1 | 1 | 1 | 1 | 1 | 1 | 1 | 5^b^ |
| [33] | J. Pearson | 1 | 1 | 1 | 1 | 0 | 0 | 0 | 2^e^ |
| [34] | K. L. Bennell | 1 | 1 | 1 | 1 | 1 | 1 | 1 | 5^b^ |
| [11] | K. L. Bennell | 1 | 1 | 1 | 1 | 1 | 1 | 1 | 5^b^ |
| [35] | L. C. Li | 1 | 1 | 1 | 0 | 1 | 1 | 1 | 4^b^ |
| [36] | Belinda J Lawford | 1 | 1 | 1 | 1 | 1 | 1 | 1 | 5^b^ |
| [37] | K. Button | 1 | 1 | 1 | 1 | 1 | 1 | 0 | 4^e^ |
| [38] | G. Mecklenburg | 1 | 1 | 1 | 1 | 1 | 1 | 1 | 5^b^ |
| [39] | P. W. Kline | 1 | 0 | 1 | 1 | 0 | 1 | 0 | - |
| [40] | R. K. Nelligan | 1 | 1 | 1 | 1 | 1 | 1 | 1 | 5^a^ |
| [41] | T. Pelle | 1 | 1 | 1 | 1 | 1 | 1 | 1 | 5^b^ |
| [42] | J. F. Bailey | 1 | 1 | 1 | 1 | 1 | 1 | 0 | 4^b^ |
| [43] | K. Baker | 1 | 1 | 1 | 0 | 1 | 1 | 1 | 4^b^ |
| [33] | Kim Bennell | 1 | 1 | 1 | 1 | 1 | 1 | 1 | 5^b^ |
| [44] | M. L. Fitzgibbon | 1 | 1 | 1 | 0 | 1 | 1 | 1 | 4^a^ |
| [45] | R. S. Hinman | 1 | 1 | 1 | 1 | 1 | 1 | 1 | 5^b^ |
| [46] | R. S. Hinman | 1 | 0 | 1 | 1 | 0 | 1 | 0 | - |
| [47] | L. C. Li | 1 | 1 | 1 | 0 | 1 | 1 | 1 | 4^b^ |
| [48] | R. K. Nelligan | 1 | 1 | 1 | 1 | 0 | 0 | 0 | 2^a^ |
| [49] | E. Dunphy | 1 | 1 | 1 | 1 | 1 | 1 | 1 | 5^b^ |
| [50] | M. F. Lindberg | 1 | 0 | 1 | 1 | 0 | 1 | 0 | - |
| [21] | R. Nelligan | 1 | 1 | 1 | 1 | 1 | 1 | 1 | 5^b^ |
| [51] | T. Pelle | 1 | 1 | 1 | 1 | 1 | 0 | 1 | 4^d^ |
| [52] | T. Rognsvåg | 1 | 1 | 1 | 1 | 0 | 1 | 0 | 3^a^ |
| [53] | K. L. Bennell | 1 | 0 | 1 | 1 | 0 | 1 | 0 | - |
| [54] | D. Groves Williams | 1 | 0 | 1 | 1 | 0 | 1 | 0 | - |
| [55] | R. S. Hinman | 1 | 0 | 1 | 1 | 0 | 1 | 0 | - |
| [56] | E. Östlind | 1 | 1 | 1 | 1 | 0 | 1 | 1 | 4^a^ |
| [57] | E. Östlind | 1 | 1 | 1 | 0 | 1 | 0 | 1 | 3^b^ |
| [58] | J. L. Whittaker | 1 | 1 | 0 | 1 | 1 | 1 | 1 | 4^c^ |
| [59] | K. Godziuk | 1 | 1 | 1 | 1 | 1 | 0 | 0 | 3^c^ |
| [60] | N. Lorbeer | 1 | 1 | 1 | 1 | 1 | 0 | 1 | 4^b^ |
| [61] | J. K. Scheer | 1 | 1 | 1 | 1 | 1 | 0 | 1 | 4^c^ |
| [62] | L. K. Truong | 1 | 1 | 1 | 1 | 1 | 1 | 1 | 5^a^ |
| [63] | F. Weber | 1 | 0 | 1 | 1 | 0 | 1 | 0 | - |
| a. Qualitative b. Quantitative randomized controlled trials c. Quantitative non-randomized  d. Quantitative descriptive e. Mixed methods - (not applicable) | | | | | | | | | |
